# CR-YOLOv9: Improved YOLOv9 Multi-Stage Strawberry Fruit Maturity Detection Application Integrated with CRNET

**DOI:** 10.3390/foods13162571

**Published:** 2024-08-17

**Authors:** Rong Ye, Guoqi Shao, Quan Gao, Hongrui Zhang, Tong Li

**Affiliations:** 1 College of Food Science and Technology, Yunnan Agricultural University, Kunming 650201, China; 15912913557@163.com; 2 The Key Laboratory for Crop Production and Smart Agriculture of Yunnan Province, Yunnan Agricultural University, Kunming 650201, China; 15751769522@163.com; 3 College of Big Data, Yunnan Agricultural University, Kunming 650201, China; gaoq@ynau.edu.cn; 4 College of Plant Protection, Yunnan Agricultural University, Kunming 650201, China; hongruizh@126.com

**Keywords:** target detection, YOLOv9, maturity classification, CGA, CRNet, Shape-IoU

## Abstract

Strawberries are a commonly used agricultural product in the food industry. In the traditional production model, labor costs are high, and extensive picking techniques can result in food safety issues, like poor taste and fruit rot. In response to the existing challenges of low detection accuracy and slow detection speed in the assessment of strawberry fruit maturity in orchards, a CR-YOLOv9 multi-stage method for strawberry fruit maturity detection was introduced. The composite thinning network, CRNet, is utilized for target fusion, employing multi-branch blocks to enhance images by restoring high-frequency details. To address the issue of low computational efficiency in the multi-head self-attention (MHSA) model due to redundant attention heads, the design concept of CGA is introduced. This concept aligns input feature grouping with the number of attention heads, offering the distinct segmentation of complete features for each attention head, thereby reducing computational redundancy. A hybrid operator, ACmix, is proposed to enhance the efficiency of image classification and target detection. Additionally, the Inner-IoU concept, in conjunction with Shape-IoU, is introduced to replace the original loss function, thereby enhancing the accuracy of detecting small targets in complex scenes. The experimental results demonstrate that CR-YOLOv9 achieves a precision rate of 97.52%, a recall rate of 95.34%, and an mAP@50 of 97.95%. These values are notably higher than those of YOLOv9 by 4.2%, 5.07%, and 3.34%. Furthermore, the detection speed of CR-YOLOv9 is 84, making it suitable for the real-time detection of strawberry ripeness in orchards. The results demonstrate that the CR-YOLOv9 algorithm discussed in this study exhibits high detection accuracy and rapid detection speed. This enables more efficient and automated strawberry picking, meeting the public’s requirements for food safety.

## 1. Introduction

Ripe strawberries are characterized by a delightful blend of sourness, sweetness, and juiciness, while also being rich in essential dietary components, such as vitamins, minerals, folic acid, and fiber [1]. In China, strawberries are highly favored and widely cultivated, with the country being the world’s largest producer of this fruit. The ripening period of strawberries typically spans three to four months, during which the nutrient composition varies based on the ripeness of the fruit. If strawberries are harvested too early, not only will their nutritional value be low, but the taste and quality will also suffer. On the other hand, if they are harvested too late, the fruits are more likely to rot, impacting transportation and storage, and potentially leading to food safety concerns. Currently, strawberry harvesting is predominantly based on the manual observation of their growth status to determine the optimal time for picking. This traditional method is not only inefficient and costly, but also fails to meet the demands of modern industrialization [2]. In light of the dwindling agricultural labor force, performing an intelligent and automated detection of strawberry fruits in intricate orchard settings holds significant practical importance. Therefore, researching an efficient, accurate, and suitable strawberry ripeness detection system for orchard deployment will not only provide a valuable reference for modernizing the strawberry industry, but also meet the public’s demand for food safety.

Strawberry ripeness detection falls under the category of target detection [3,4,5]. As artificial intelligence technology continues to advance, target detection algorithms are also evolving and being applied in various areas of food detection [6,7,8], such as meat freshness detection [9,10], fruit maturity detection [11,12,13,14], and food classification [15,16]. Target detection algorithms are typically categorized into two groups: single-stage target detection algorithms [17], such as the YOLO series [18] and SSD [19]; and two-stage target detection algorithms [20], such as R-CNN [21], Fast R-CNN [22], and Faster R-CNN [23]. The YOLO series model is currently the predominant single-stage target detection algorithm and has found extensive application in fruit maturity detection. In their study, Wang et al. [24] introduced the DSE-YOLO model specifically designed for detecting small strawberry targets. The model incorporates pointwise convolution and dilated convolution to capture detailed information and semantic features in both horizontal and vertical dimensions, enabling the accurate detection of various stages of strawberry fruit growth in natural environments. Building upon this work, An et al. [25] further enhanced the spatial interaction capabilities and detection accuracy of small target fruits by refining the YOLOX model. Their improvements led to the successful monitoring of strawberry fruits across five distinct growth stages. Cuong et al. [26] utilized technology based on the YOLOv4 model for real-time monitoring on mobile devices, achieving a recognition accuracy of 98.26% on the pineapple dataset. To enable the multi-task detection of cherry tomatoes, Chen et al. [27] incorporated two additional decoders to enhance the YOLOv7 loss function, resulting in the development of the MTD-YOLOv7 model with improved generalization capabilities. Li et al. [28] leveraged the MHSA mechanism to enhance the backbone network of YOLOv8, boosting the network’s capacity to extract diverse features and demonstrating a strong performance for tomato maturity grading and counting. The DSConv module was integrated into the YOLOv8 network by You et al. [29], resulting in a reduction in parameters. Additionally, a spatial attention mechanism was incorporated into the feature fusion network to enhance the network’s feature expression capability, leading to an average accuracy of 98.6%. On the other hand, Yang et al. [30] combined a Swin-Transformer with the YOLOv8s model, resulting in a more efficient feature fusion network and a 0.5% increase in the detection accuracy compared to the original model.

The main contributions of this article are as follows:

(1) The composite refinement network (CRNet) is proposed to utilize multi-branch blocks for target fusion in order to restore high-frequency details in images and achieve complete image restoration and enhancement. Additionally, it incorporates convolution enhancement blocks, large kernel convolution, and ConvFFN to expand the receptive field and improve feature fusion capabilities. This addresses the constraints posed by sensors and other equipment in real-world scenarios, allowing for the enhancement of low-dynamic images with blur and noise. These advancements provide a technical groundwork for the development of one-step strawberry ripeness image detection.

(2) In order to address the issue of low computational efficiency resulting from redundant attention heads in multi-head self-attention (MHSA) mechanism, this paper proposes the concept of CGA. CGA aligns input feature grouping with the number of attention heads, providing each attention head with distinct segmentations of complete features to explicitly distribute attention calculations across the heads. Additionally, a feed-forward network (FFN) is introduced. This attention mechanism utilizes global embedding to maintain an optimal performance while dividing multiple attention heads through feature grouping to enhance memory storage efficiency. Finally, intra-group feature dimensionality reduction is employed to reduce computational redundancy.

(3) A hybrid operator is proposed to enhance the restoration of original detailed features in small target features without adding excessive parameters during the upsampling process. This approach aims to reduce information loss due to channel compression, while also improving computational efficiency for image classification and target detection. The operator achieves this by sharing self-attention and convolution modules.

(4) The current rapid progress in bounding box regression (BBR) is primarily achieved through the addition of new loss terms. The IoU cannot be dynamically adjusted based on varying detection model performance and different detection tasks. To enhance the detection of small strawberries, the concept of Inner-IoU is introduced and integrated with Shape-IoU (Shape Intersection over Union) to replace the original loss function. This adjustment allows the model to prioritize the recognition of overlapping detection frames, thereby enhancing the accuracy of small target detection in complex scenarios.

## 2. Materials and Methods

### 2.1. Data Acquisition

In accordance with the standard NY/T 1789-2009 ‘Strawberry Grade Specifications Part 3′, the quality of strawberries is typically assessed based on criteria such as the absence of rot or deterioration, color maturity, lack of mechanical damage, freshness of sepals and fruit stems, absence of pests and diseases, and absence of abnormalities. The ripening process of strawberries is categorized into four stages—green ripening period, white ripening period, color-changed period, and red ripening period—based on external moisture and other factors. The classification criteria can be found in Table 1.

**Figure 1:**
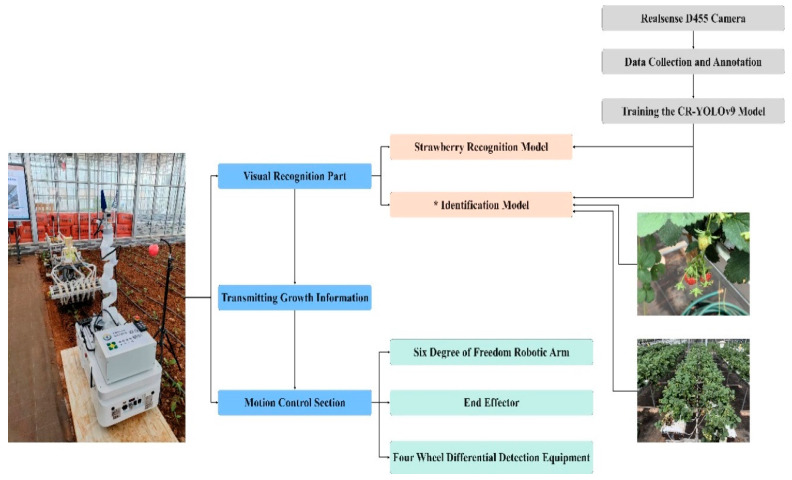
Intelligent equipment design process diagram. * stands for Mi model.

**Figure 2:**
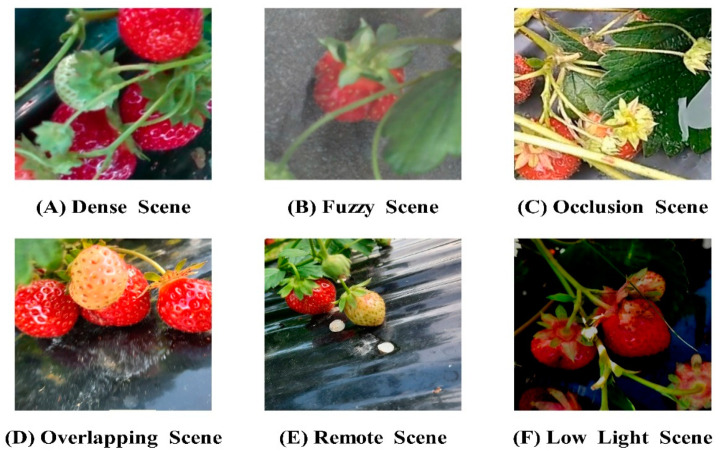
Partial image of the dataset.

The experimental research area was located at the coordinates 102°75′ N, 25°13′ E, based on the Yunnan Provincial Key Laboratory of Crop Production and Smart Agriculture, in conjunction with the strawberry planting experimental base of the Yunnan Provincial Dian-Taiwan Characteristic Agricultural Industrialization Engineering Research Center. This study focuses on ‘Red Face’, known for its high yield, ornamental value, good fruit quality, and strong flavor, making it of significant research interest. The collection equipment utilized a RealSense D455 depth industrial camera model with an 8 mm lens. A ring-light source was positioned above the field of view, and the entire setup was secured with a high-precision fine-adjustable bracket. A black curtain served as the background for the images. The image acquisition device and process are detailed in Figure 1. To evaluate the algorithm presented in this article, it is recommended to select diverse images, such as dense scenes, blurred scenes due to exposure, occlusion scenes, scenes with multiple overlapping targets, long-distance scenes, and scenes with backlight shadows. These images will provide a comprehensive assessment of the algorithm’s performance. Utilize the open source annotation tool LabelImg to manually annotate the collected strawberry images and save the resulting *.xml format files in a designated folder, as depicted in Figure 2.

#### Classification of Different Strawberry Maturity Levels

**Figure 3:**
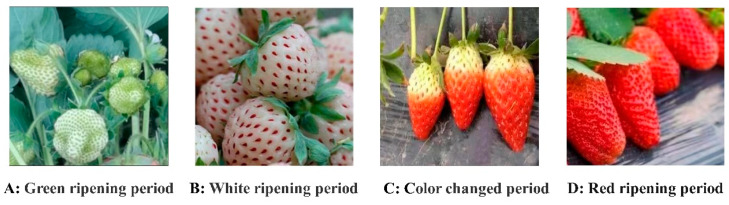
Strawberry ripeness classification.

Conventional methods for classifying strawberry maturity typically categorize the fruit into the red ripening period, color-changed period, white ripening period, and green ripening period, based on the extent of coloration on the fruit’s surface. The colored areas represent approximately 100%, 75%, 50%, and 25% of the fruit’s surface. Given the challenges of accurately assessing strawberry peel coloration in natural settings, this study proposes a classification system based on the peel coloration area. The four levels identified are: red ripening period (full maturity), characterized by a fully red peel; color-changed period (maturity), where the peel shows alternating red and green colors; white ripening period (immaturity), with most of the fruit’s surface being white; and green ripening period (immaturity), where the peel is predominantly green. Each type of strawberry is labeled accordingly, as illustrated in Figure 3.

### 2.2. Composite Refinement Network

Real greenhouse shooting scenes often face challenges, such as poor lighting conditions, foggy environments, long exposure times, and sensor limitations. These factors can lead to image degradation, including reduced visibility, high levels of noise, and artifacts, all of which can significantly impact target detection accuracy. To address these issues, researchers have explored various deblurring, denoising, and HDR imaging techniques. Current methods often focus on specific individual tasks, resulting in unsatisfactory images. To address this issue, it is essential to develop a model capable of simultaneously handling image restoration and enhancement tasks. One common approach to improving detection performance in low-light scenes involves integrating an image enhancement algorithm before the detection algorithm to restore details and texture to low-quality images to achieve normal lighting effects. Traditional image enhancement algorithms typically include those based on histogram equalization [31,32] and the Retinex theory [33,34,35].

CRNet utilizes a pooling layer to effectively distinguish between high-frequency and low-frequency information, and employs multi-branch blocks for fusion to address the issue of inadequate high-frequency details in image restoration tasks. To enhance the integration of various image features, CRNet incorporates a convolutional enhancement block, which is a convolution module primarily consisting of large kernel convolutions to expand the model’s receptive field. Additionally, ConvFFN with a reverse bottleneck structure is utilized for comprehensive feature fusion.

#### 2.2.1. Overview of CRNet

A series of original images $\left\{R_{1},R_{2},\ldots ,R_{N}\right\}$ captured in a dynamic task scene with varying exposure levels was processed through denoising, deblurring, and HDR reconstruction simultaneously. The process involved selecting five original images $\left\{R_{1},R_{2},\ldots ,R_{N}\right\}$ sorted from lowest to highest exposure, with $R_{1}$ as the reference. Each image, $R_{i}$, is then normalized to $\frac{R_{i}}{\Delta t_{i}/\Delta t_{1}}$, where $\Delta t_{i}$ represents the exposure time of the *i*-th image. Following the multi-exposure HDR reconstruction method [36,37], the normalized original image undergoes gamma mapping and conversion to generate $\left\{L_{1},L_{2},L_{3},L_{4},L_{5}\right\}$, which is expressed as:

(1) $$L_{i}={\left(\frac{R_{i}}{\Delta t_{i}/\Delta t_{1}}\right)}^{\gamma }$$

Among them, γ represents the gamma correction parameter, usually set to 0.45.

Subsequently, connect each $L_{i}$ with its corresponding $R_{i}$ according to the following equation to form $\left\{I_{1},I_{2},I_{3},I_{4},I_{5}\right\}$:

(2) $$I_{i}=\left\{\frac{R_{i}}{\Delta t_{i}/\Delta t_{1}},L_{i}\right\}$$

Then, $\left\{I_{1},I_{2},I_{3},I_{4},I_{5}\right\}$ were input into the model, and a noise free and blurred HDR image was derived based on the following equation, represented as:

(3) $$\hat{H}=f\left(I_{1},I_{2},I_{3},I_{4},I_{5};\theta \right)$$

where the function f(⋅) represents the imaging network and θ represents the network parameters.

#### 2.2.2. Frequence Separation and Fusion

**Figure 4:**
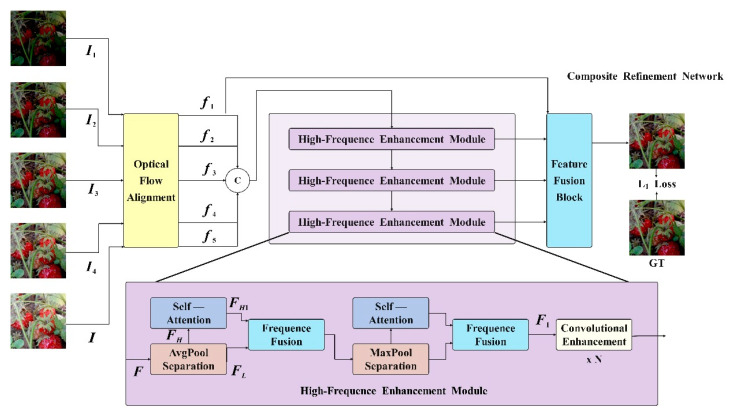
An overview of the CRNet model.

**Figure 5:**
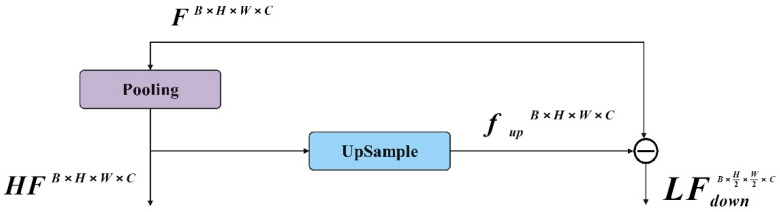
The pooling layer effectively separates high- and low-frequency information.

In the unified task of image restoration and enhancement, the enhancement of high-frequency feature information is very important. It is an effective method to amplify the high-frequency feature and the low-frequency feature. The CRNET model is shown in Figure 4. The traditional separation technology needs a lot of computational overheads and cannot directly integrate the network. In order to minimize the computational cost associated with separating high-frequency and low-frequency features, a pool layer is used, as shown in Figure 5. In CRNET, average pooling and maximum pooling are used. Specifically, the pooling layer downsamples the input feature map of dimension B×H×W×C to reduce the resolution, $F_{L}^{B\times \frac{H}{2}\times \frac{W}{2}\times C}$, in order to obtain low-frequency features, $F_{L}$. These features are then upsampled to the original dimensions, $f_{up}^{B\times H\times W\times C}$, using bilinear interpolation. The high-frequency features, $F_{H}$ of F, are then calculated by subtracting $F_{L}$ from the original features, F. This method efficiently captures both the high- and low-frequency features of the image, as demonstrated in the following formula:

(4) $$F_{L}=Pooling(F)$$

(5) $$F_{H}=F-\mathrm{Upsample}(F_{L})$$

where Pooling represents the downsampling operation of the pooling layer, and Upsample represents the upsampling operation of bilinear interpolation.

**Figure 6:**
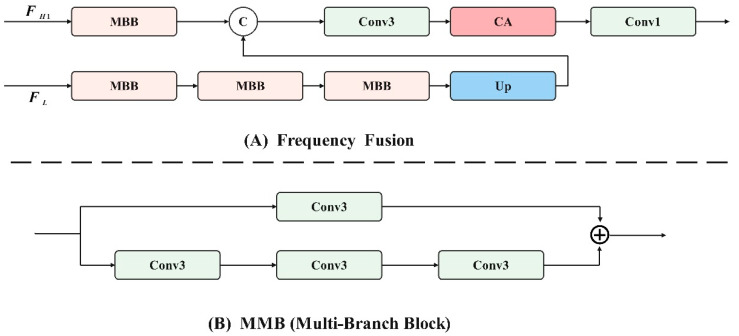
Asymmetric parallel convolutional groups.

After obtaining the clearly extracted high-frequency features, $F_{H}$, the self-attention mechanism is utilized to globally enhance them and obtain $F_{H_{1}}$. To address issues of information loss and insufficient fusion during the fusion process, multi-branch blocks are employed, as illustrated in Figure 6A. These blocks comprise dual-path convolutional components, each path using a different number of convolutions. The first branch incorporates three convolution kernels, focusing on high-frequency image details, while the second branch includes only one convolution kernel, emphasizing low-frequency content and texture details. This approach effectively combines high- and low-frequency features. The specific formula is as follows:

(6) $$H=MBB\left(F_{H1}\right)$$

(7) $$L=MBB\left(MBB\left(MBB\left(F_{L}\right)\right)\right)$$

(8) $$Out={Conv}_{1\times 1}(CA({Conv}_{3\times 3}(Concatenate(Up(L),H))))$$

Among them, MBB represents multi-branch blocks, Conv1 represents 1 × 1 convolution, Conv3 represents 3 × 3 convolution, CA represents channel attention, and Up represents bilinear interpolation upsampling.

#### 2.2.3. Convolutional Enhancement

**Figure 7:**
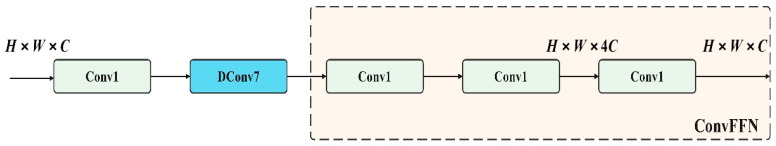
Convolutional enhancement block.

In order to enhance the receptive field and effectively integrate input features, convolutional enhancement blocks are utilized in the network, as illustrated in Figure 7. The convolution module employs 7 × 7 depthwise separable convolutions to achieve a broad receptive field and incorporates an inverse bottleneck structure, ConvFFN, for comprehensive information extraction. Furthermore, it serves as a high-pass filter, facilitating the efficient fusion of content from the five input frames and implicitly boosting high-frequency information. The feed-forward network (FFN) is introduced to enhance the performance of the attention mechanism by adding non-linearity, refining feature representations, increasing network depth, and improving robustness and generalization. It helps balance the computational load, mitigate overfitting, and supports the sequential refinement of features. These enhancements ensure that the model can capture complex patterns, generalize well to unseen data, and maintain a high performance in real-time applications, such as strawberry ripeness detection. The specific formula is outlined as follows:

(9) $$F_{2}=\mathrm{ConvFFN}\left({\mathrm{Conv}}_{1\times 1}\left({\mathrm{DConv}}_{7\times 7}\left({\mathrm{Conv}}_{1\times 1}\left(F_{1}\right)\right)\right)\right)$$

Among them, ${\mathrm{Conv}}_{1\times 1}$ represents a 1 × 1 convolution, and ${\mathrm{DConv}}_{7\times 7}$ represents the depthwise separable 7 × 7 convolution.

**Figure 8:**
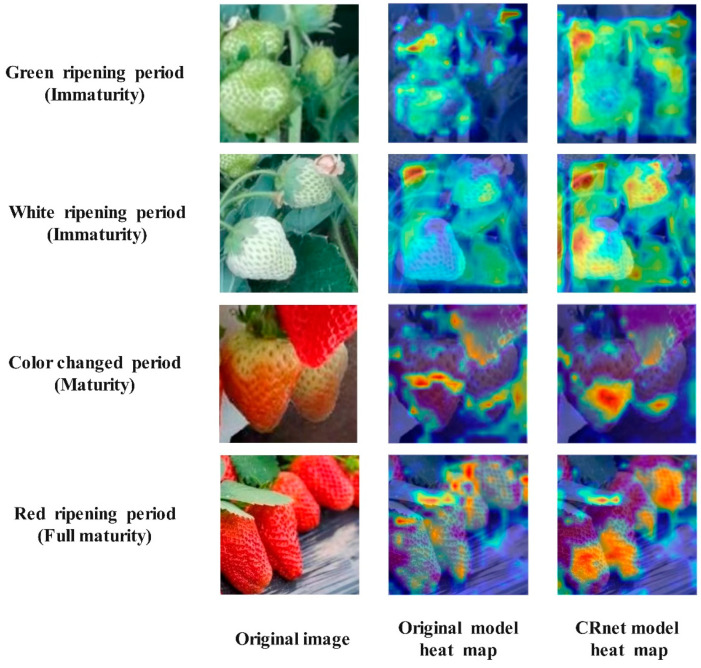
Heat maps of strawberry fruits at different stages.

To delve deeper into the impact of CRNet and gain a clearer understanding of its role in detecting strawberry ripeness, the Grad-CAM heat map visualization technique emphasizes the model’s ability to identify the maturity level of strawberries by analyzing the weight of the ‘maturity’ category in the final convolution layer. The visualization results, as depicted in Figure 8, reveal that the model’s visual analysis in this study significantly influences the decision-making process, with a stronger focus on mature areas. This concentrated attention on mature regions underscores the model’s effectiveness in detecting strawberry ripeness.

### 2.3. YOLOv9 Model

#### 2.3.1. Improving the YOLOv9 Model

**Figure 9:**
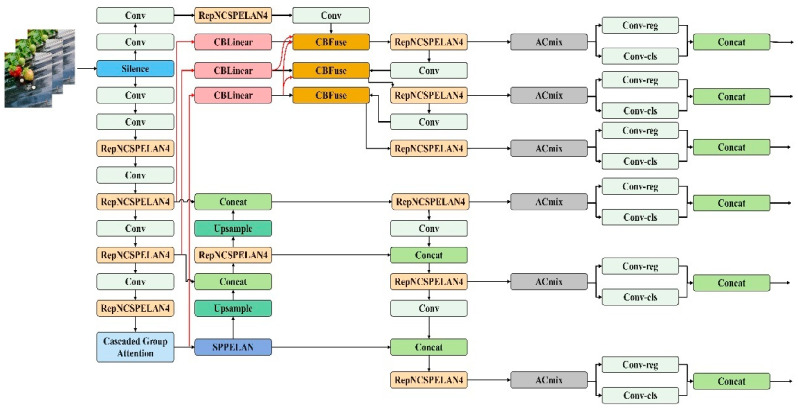
Structure of the improved YOLOv9 algorithm.

YOLOv9, the most recent iteration of the YOLO (You Only Look Once) series, introduces advancements to real-time target detection systems. Built upon YOLOv7, it leverages the General ELAN (GELAN) architecture and programmable gradient information (PGI) to enhance both the efficiency and accuracy of target detection. The utilization efficiency of model parameters is notably improved. Thus, in this research, YOLOv9 is employed for detecting strawberry maturity, as illustrated in Figure 9.

#### 2.3.2. Efficient Vision Transformer with Cascaded Group Attention

**Figure 10:**
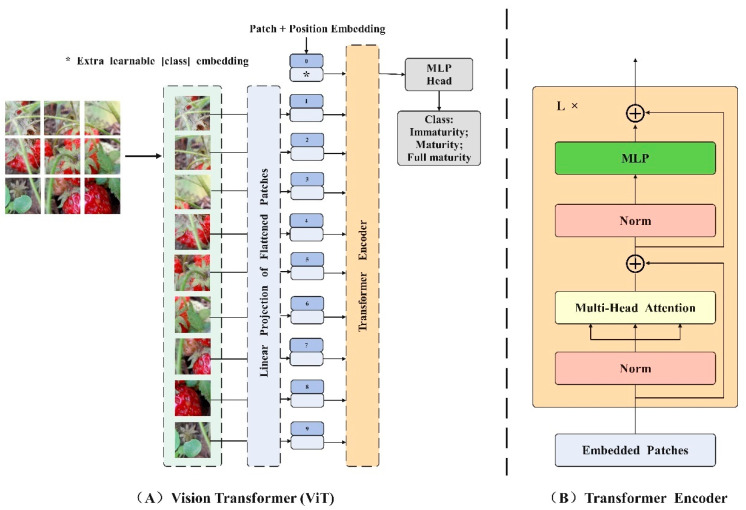
Vision Transformer network structure.

Vision Transformer (ViT) is a groundbreaking approach that leverages Transformer architecture for image classification by tokenizing and flattening images into token sequences. This method involves designing a more suitable backbone network based on Transformer architecture for computer vision tasks. The Transformer model, a seminal work in natural language processing (NLP) introduced by Google in 2017 [38], incorporates a self-attention mechanism to enable a global understanding of images, establish feature dependencies, and leverage contextual information for parallel training. Although the Image Transformer model from 2018 applies Transformer to image classification, it still relies on convolution operations and does not fully exploit the benefits of the self-attention mechanism [39]. Until 2020, Dosoviskiy et al. introduced the ViT model, which was the first to apply the original Transformer model to image classification tasks. To enable the Transformer structure to process images, the ViT model introduces the concept of image patches (Patch). These image patches are transformed into sequence data through linear projection and position coding before being input into the Transformer. Additionally, a classification flag (Class) is added before the sequence data in order to better capture global information. The self-attention mechanism in each layer of the Transformer allows for capturing dependencies between image features and leveraging contextual information for a comprehensive global understanding. Following the self-attention mechanism, the output of the Transformer passes through a fully connected layer and a SoftMax layer to generate the final image classification result. The complete structure of the Vision Transformer model can be observed in Figure 10, which comprises three main modules: the embedding layer, the Transformer encoder, and the MLP head.

**Figure 11:**
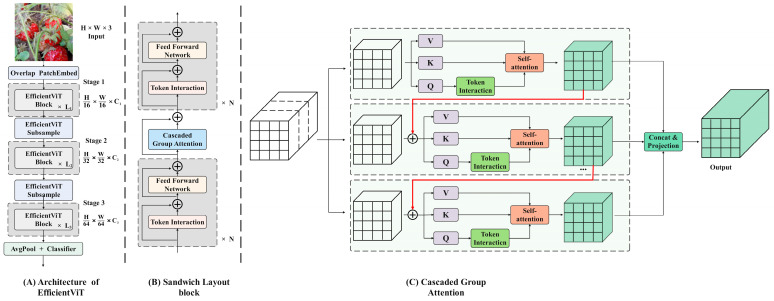
Overview of EfficientViT. (**A**) Architecture of EfficientViT; (**B**) sandwich layout block; (**C**) cascaded group attention.

Due to the local perceptual characteristics of the convolution operation, the overall correlation between the data can be overlooked, leading to deviations in understanding the overall semantic information. To enhance the multi-scale representation ability of images, various aspects, such as different ideas, advantages, limitations, and complexity of the improved model, are integrated. This section introduces a new hierarchical model named EfficientViT, designed for fast inference. The framework is illustrated in Figure 11, with the adoption of a new efficient visual converter building block depicted in Figure 11B. This module incorporates a memory-efficient sandwich layout, a cascaded group attention module, and a parameter redistribution strategy to enhance model efficiency in terms of memory, computation, and parameters. It utilizes self-attention layers that are less memory-constrained and more memory-efficient feed-forward network (FFN) layers for channel communication. Spatial mixing is achieved by a single self-attention layer, $\Phi_{i}^{A}$, sandwiched between FFN layers, $\Phi_{i}^{F}$, calculated as follows:

(10) $$X_{i+1}=\overset{N}{\prod }\;\Phi_{i}^{F}(\Phi_{i}^{A}(\overset{N}{\prod }\;\Phi_{i}^{F}(X_{i})))$$

Among them, $X_{i}$ is the complete input feature of the i-th block. The block transforms $X_{i}$ into $X_{i+1}$, with N FFNs before and after a single self-attention layer. This design can reduce the memory time consumption caused by the self-attention layer and adopt more FFN layers to achieve efficient communication between different feature channels. In addition, deep convolution (DWConv) applies an additional token interaction layer before each FFN, introducing the inductive bias of local structural information to enhance the model’s ability.

Header redundancy in the MHSA model is a significant issue that can result in computational inefficiency. The implementation of cascaded group attention (CGA) offers various segmentations of complete features for each head, effectively breaking down the attention calculation across heads. This can be expressed as:

(11) $${\tilde{X}}_{ij}=Atten(X_{ij}W_{ij}^{Q},X_{ij}W_{ij}^{K},X_{ij}W_{ij}^{V})$$

(12) $${\tilde{X}}_{i+1}=Concat[{\tilde{X}}_{ij}{]}_{j=1:h}W_{i}^{P}$$

Among them, the j-th head calculates the self-attention layer on $X_{ij}$; $X_{ij}$ is the j-th segmentation of the input feature $X_{i}$, that is, $X_{i}=[X_{i1},X_{i2},\ldots ,X_{ih}]$, 1≤j≤h. h is the total number of heads, $W_{ij}^{Q}$, $W_{ij}^{K}$, and $W_{ij}^{V}$ are the projection layers that divide the input feature into different subspaces, and $W_{i}^{P}$ is the dimensional linear layer that projects the connected output features consistent with the input.

While utilizing feature segmentations instead of full features for each head is more efficient and reduces computational overheads, the capacity of the model can still be enhanced by learning projections of features with more information for the Q, K, and V layers. The attention map of each head is calculated in a cascade manner, as illustrated in Figure 11C, where the output of each head is added to subsequent heads to iteratively enhance feature representation.

(13) $$X_{ij}^{’}=X_{ij}+{\tilde{X}}_{i(j-1)},\;\begin{array}{c} 1<j\leq h \end{array}$$

where $X_{ij}^{’}$ is the addition of the j-th input division, $X_{ij}$, and the (j−1) head output ${\tilde{X}}_{i(j-1)}$ calculated by Equations (11) and (12). When calculating the self-attention value, it replaces $X_{ij}$ as the new input feature of the jth header. In addition, a token interaction layer is added after the Q projection to enable the self-attention mechanism to jointly capture local and global relationships and further enhance feature representation.

#### 2.3.3. Self-Attention and Convolution Mechanisms

Convolutional neural networks leverage convolution kernels to extract local features and have emerged as a dominant and conventional technology in a wide range of visual tasks. Theoretical analysis indicates that, with sufficient capacity, the self-attention mechanism has the capability to represent the function class of any convolutional layer. As a result, recent research endeavors have delved into the potential of integrating self-attention mechanisms in visual tasks. Two primary approaches have been explored: one involves utilizing the self-attention mechanism as a fundamental component of the network, while the other entails combining the self-attention mechanism with the convolution network as a supplementary element. A hybrid operator enhances the restoration of original detailed features from small target features during the upsampling process by combining self-attention and convolution mechanisms. This approach ensures detailed feature restoration without adding excessive parameters by leveraging the strengths of both techniques: the self-attention mechanism captures global dependencies and context, while the convolution network efficiently handles local patterns and fine details. The integration of these methods allows for precise upsampling with minimal parameter overheads, ensuring high-quality feature restoration for small targets.

**Figure 12:**
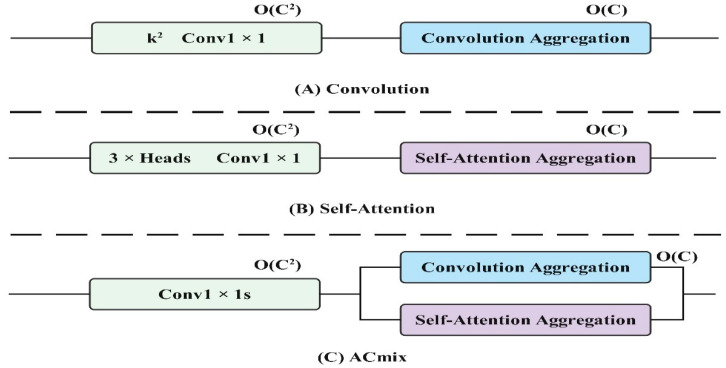
Convolution combined with the self-attention mechanism.

Visual Transformers [40] have shown significant advancements in computer vision tasks, with the research focusing on enhancing Transformer models with convolution operations to introduce additional inductive bias. The CvT [41] incorporates a convolution network during tokenization and utilizes strided convolution to reduce self-attention computational complexity. As shown in Figure 12A. The ViT [42] with the convolutional stem suggests adding convolution to the early stages for more stable training. The CSwin Transformer [43] leverages convolution-based position encoding to enhance downstream tasks. As shown in Figure 12B. Conformer [44] merges Transformer with an independent CNN model for a combined approach. As shown in Figure 12C.

**Figure 13:**
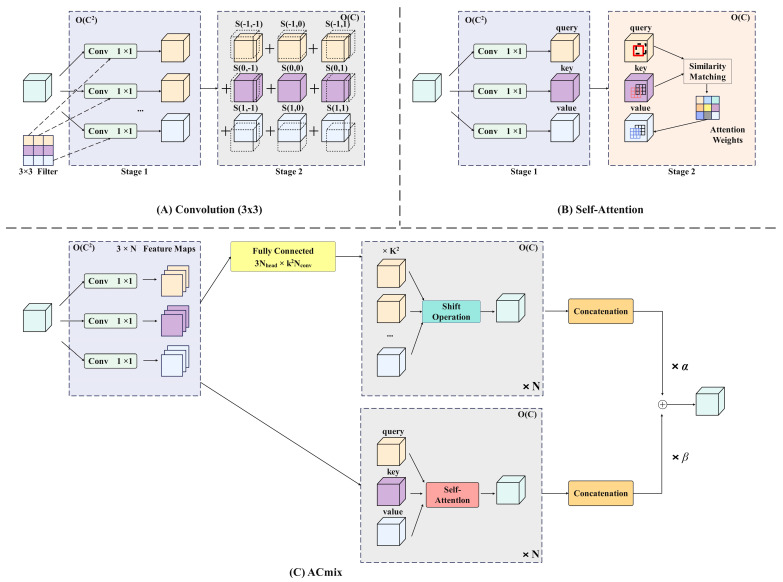
Schematic diagram of the self-attention and convolution hybrid module.

As shown in Figure 13A, assuming that the stride of the convolution is 1, consider a standard convolution with the kernel $K\in R^{C_{out}\times C_{in}\times k\times k}$, where K is the convolution kernel size, and $C_{in}$ and $C_{out}$ are the sizes of the input and output channels, respectively. Given tensor $F\in R^{C_{in}\times H\times W}$, $G\in R^{C_{out}\times H\times W}$ is the input and output feature map, where H, W represent the height and width, respectively, with $f_{ij}\in R^{C_{in}}$, $g_{ij}\in R^{C_{out}}$ as pixels (i,j) corresponding to F and G, respectively. Then standard convolution can be expressed as:

(14) $$g_{ij}=\sum_{p,q}\;K_{p,q}f_{i+p-\lfloor k/2\rfloor ,j+q-\lfloor k/2\rfloor }$$

where $K_{p,q}\in R^{C_{out}\times C_{in}}$, p,q∈{0,1,…,k−1} denote the positions relative to the nucleus (p,q). Finally, Equation (14) can be rewritten as the sum of feature maps at different core locations:

(15) $$g_{ij}=\sum_{p,q}\;g_{ij}^{\left(p,q\right)}$$

(16) $$g_{ij}^{\left(p,q\right)}=K_{p,q}f_{i+p-\lfloor k/2\rfloor ,j+q-\lfloor k/2\rfloor }$$

Furthermore, Shift operations, $\tilde{f}≜\mathrm{Shift}(f,\Delta x,\Delta y)$, include:

(17) $${\tilde{f}}_{i,j}=f_{i+\Delta x,j+\Delta y},\forall i,j$$

where Δx, Δy are the horizontal displacement and vertical displacement, respectively. Then, Equation (16) can be rewritten as:

(18) $$g_{ij}^{\left(p,q\right)}=K_{p,q}f_{i+p-\lfloor k/2\rfloor ,j+q-\lfloor k/2\rfloor }=\mathrm{Shift}(K_{p,q}f_{ij},p-\lfloor k/2\rfloor ,q-\lfloor k/2\rfloor )$$

Therefore, the standard convolution can be summarized into two stages:

(19) $$\mathrm{Stage}\;I:\;{\tilde{g}}_{ij}^{\left(p,q\right)}=K_{p,q}f_{ij}$$

(20) $$\mathrm{Stage}\;\mathrm{II}:\;g_{ij}^{\left(p,q\right)}=\mathrm{Shift}({\tilde{g}}_{ij}^{\left(p,q\right)},p-\lfloor k/2\rfloor ,q-\lfloor k/2\rfloor )$$

(21) $$g_{ij}=\sum_{p,q}\;g_{ij}^{\left(p,q\right)}$$

#### 2.3.4. Self-Attention Mechanism

Attention mechanisms are commonly used for visual tasks to enable models to concentrate on crucial regions within a broader context, surpassing the limitations of traditional convolutions. This concept is illustrated in Figure 13B.

Let $F\in R^{C_{in}\times H\times W}$ and $G\in R^{C_{out}\times H\times W}$ represent the input and output features, respectively. Define $f_{ij}\in R^{C_{in}}$ and $g_{ij}\in R^{C_{out}}$ as the pixels at positions (i,j). The output of the attention module is then calculated as:

(22) $$g_{ij}=\overset{N}{\left|\right|}\left(\sum_{a,b\in N_{k}(i,j)}\;A(W_{q}^{\left(l\right)}f_{ij},W_{k}^{\left(l\right)}f_{ab})W_{v}^{\left(l\right)}f_{ab}\right)$$

Among them, || represents the concatenation of N attention head outputs. $W_{q}^{\left(l\right)}$, $W_{k}^{\left(l\right)}$, and $W_{v}^{\left(l\right)}$ are the projection matrices of query, key, and value, respectively. $N_{k}(i,j)$ denotes a local area with a pixel space range of k centered on (i,j). $A(W_{q}^{\left(l\right)}f_{ij},W_{k}^{\left(l\right)}f_{ab})$ corresponds to the attention weight of features within $N_{k}(i,j)$.

For the self-attention module, the attention weight calculation formula is:

(23) $$A(W_{q}^{\left(l\right)}f_{ij},W_{k}^{\left(l\right)}f_{ab})=\underset{N_{k}(i,j)}{softmax}\left(\frac{\left(W_{q}^{\left(l\right)}f_{ij}{)}^{T}(W_{k}^{\left(l\right)}f_{ab}\right)}{\sqrt{d}}\right)$$

where d is the characteristic dimension of $W_{q}^{\left(l\right)}f_{ij}$.

The multi-head self-attention module can also be divided into two stages and expressed as follows:

(24) $$\mathrm{Stage}\;I:\;q_{ij}^{\left(l\right)}=W_{q}^{\left(l\right)}f_{ij},k_{ij}^{\left(l\right)}=W_{k}^{\left(l\right)}f_{ij},v_{ij}^{\left(l\right)}=W_{v}^{\left(l\right)}f_{ij}$$

(25) $$\mathrm{Stage}\;\mathrm{II}:\;g_{ij}=\begin{array}{c} N\\ \left|\right|\\ l=1 \end{array}\left(\sum_{a,b\in N_{k}(i,j)}\;A(q_{ij}^{\left(l\right)},k_{ab}^{\left(l\right)})v_{ab}^{\left(l\right)}\right)$$

The schematic diagram of the mixed self-attention and convolution module is illustrated in Figure 13C. The self-attention and convolution module reduces information loss due to channel compression by combining the global context from the self-attention mechanism with local detail preservation from the convolution network. This synergy allows the model to capture comprehensive feature information with fewer channels, thus improving the computational efficiency. The self-attention mechanism ensures that the model retains important contextual information, while the convolution network focuses on fine-grained details, together enhancing image classification and target detection accuracy without excessive computational costs.

### 2.4. Shape IoU

IoU (intersection over union) is a commonly used accuracy metric in target detection algorithms. When creating a dataset, an annotation box that is too large can result in learning excessive background information, while an annotation box that is too small can lead to incomplete feature learning. Incorrect annotations can result in learning incorrect features. The YOLO model typically uses CIoU as the bounding box regression loss function, calculated as follows:

(26) $$L_{CIoU}=1-IoU+\frac{\rho^{2}*(B,B^{gt})}{c^{2}}+\alpha *\nu$$

where $\rho^{2}*(B,B^{gt})$ is used to represent the Euclidean distance between the center point of the prediction box and the real box; $\nu =\frac{4}{\pi^{2}}({tan}^{-1}\frac{w^{gt}}{h^{gt}}-{tan}^{-1}\frac{w}{h}{)}^{2}$ is used to measure the similarity of the aspect ratio; and $\alpha =\frac{\nu }{\left(1-\mathrm{Io}U\right)+\nu }$ is used as the weight coefficient.

While CIoU enhances the accuracy of regression, it faces challenges in terms of weak generalization and slow convergence when dealing with small target objects. Reduced w and h values can disrupt the bounding box regression of CIoU, resulting in a failure to truly represent real-world cases. For instance, in the context of strawberry maturity detection, the shape of strawberries can vary significantly across different stages of growth. CIoU may be limited in dynamic growth scenarios like this, as it primarily focuses on the overlapping area of bounding boxes without taking into account the length and width. This imbalance in proportions can lead to inaccurate estimations of strawberry shapes in different samples, impacting the convergence speed of bounding boxes.

**Figure 14:**
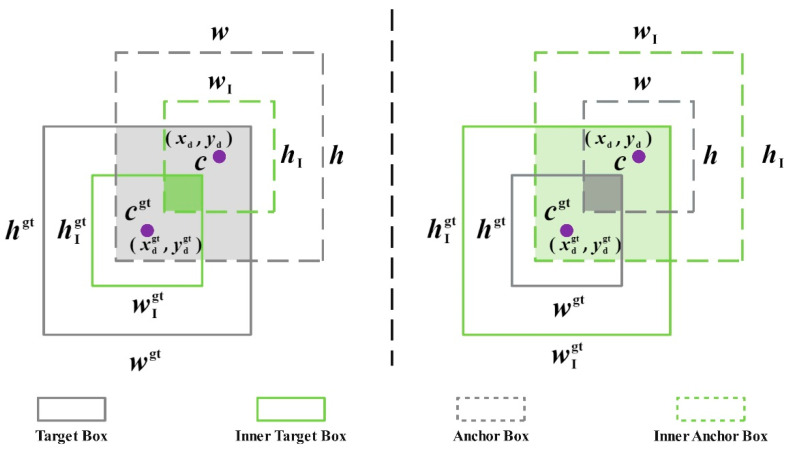
Description of Inner-IoU.

To address the aforementioned issues, a novel bounding box loss function called Inner-IoU is proposed [45]. By adjusting the scale factor ratio, the size of the secondary bounding box can be managed, leading to more precise alignments during sample positioning. Figure 14 illustrates the Inner-IoU calculation methods at smaller and larger scales.

Inner IoU introduces the scale factor ratio to control the size of the auxiliary box for loss calculations. Its definition is shown in Equations (27)–(33):

(27) $$b_{l}^{gt}=x_{c}^{gt}-\frac{w^{gt}*ratio}{2},b_{r}^{gt}=x_{c}^{gt}+\frac{w^{gt}*ratio}{2}$$

(28) $$b_{t}^{gt}=y_{c}^{gt}-\frac{h^{gt}*ratio}{2},b_{b}^{gt}=y_{c}^{gt}+\frac{h^{gt}*ratio}{2}$$

(29) $$b_{l}=x_{c}-\frac{w*ratio}{2},b_{r}=x_{c}+\frac{w*ratio}{2}$$

(30) $$b_{t}=y_{c}-\frac{h*ratio}{2},b_{b}=y_{c}+\frac{h*ratio}{2}$$

(31) $$inter=\left(min\left(b_{r}^{gt},b_{r}\right)-max\left(b_{l}^{gt},b_{l}\right)\right)*\left(min\left(b_{b}^{gt},b_{b}\right)-max\left(b_{t}^{gt},b_{t}\right)\right)$$

(32) $$union=\left(w^{gt}*h^{gt}\right)*{\left(ratio\right)}^{2}+\left(w*h\right)*{\left(ratio\right)}^{2}-inter$$

(33) $$IoU^{inner}=\frac{inter}{union}$$

where the center points of the GT box and inner GT box are represented by $\left({x_{c}}^{gt},{y_{c}}^{gt}\right)$. The center points of the prediction box and inner prediction box are represented by $\left(x_{c},y_{c}\right)$. The width and height of the real box are expressed as $w^{gt}$ and $h^{gt}$, respectively. The width and height of the prediction frame are expressed as w and h, respectively. the ratio is generally taken as [0.5, 1.5]. When the ratio < 1, the auxiliary bounding box is smaller than the actual bounding box, which can speed up the regression and convergence of high IoU samples. On the contrary, when the ratio > 1, the auxiliary bounding box is larger, which can speed up the regression process of low IoU samples.

In order to address the limitations of CIoU when dealing with the unbalanced length-to-width ratio of a strawberry-shaped bounding box, Shape-IoU [46] was introduced. This allows the model to prioritize the shape and scale of the bounding box when calculating the loss function, leading to more precise bounding box regressions. The definition of Shape-IoU is detailed in Formulas (34)–(38).

(34) $$w_{W}=\frac{2*{\left(w^{gt}\right)}^{scale}}{{\left(w^{gt}\right)}^{scale}+{\left(h^{gt}\right)}^{scale}}$$

(35) $$h_{h}=\frac{2*{\left(h^{gt}\right)}^{scale}}{{\left(w^{gt}\right)}^{scale}+{\left(h^{gt}\right)}^{scale}}$$

(36) $$distance^{shape}=\frac{h_{h}*{\left(x_{c}-x_{c}^{gt}\right)}^{2}}{c^{2}}+\frac{w_{W}*{\left(y_{c}-y_{c}^{gt}\right)}^{2}}{c^{2}}$$

(37) $$\Omega^{shape}=\sum_{t=w,h}\;{\left(1-e^{-\omega t}\right)}^{\theta },\theta =4$$

(38) $$L_{Shape-IoU}=1-IoU+distance^{shape}+0.5*\Omega^{shape}$$

where scale is the scale factor; $w_{W}$ and $h_{h}$ represent the weight coefficients in the horizontal and vertical directions, respectively.

Shape-IoU is utilized to replace the calculation component of IoU, combining the strengths of Inner-IoU and Shape-IoU to enhance the model’s generalization capability when encountering varying strawberry shapes. The enhanced Inner-ShapeIoU is presented in Equation (39).

(39) $$L_{Shape-IoU}^{inner}=1-IoU^{inner}+distance^{shape}+0.5*\Omega^{shape}$$

## 3. Results and Discussion

### 3.1. Experimental Dataset and Experimental Environment

**Figure 15:**
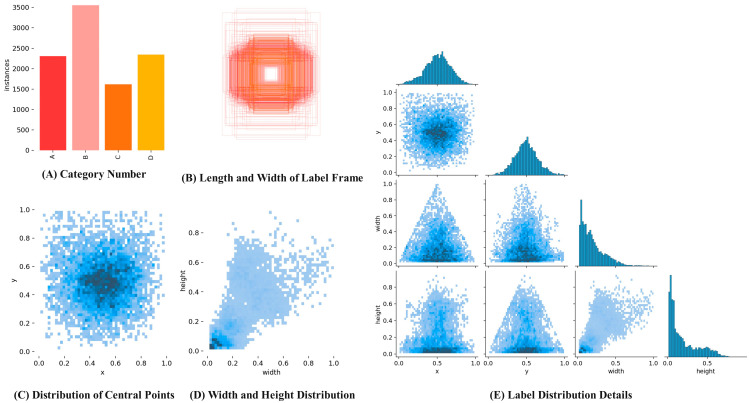
Strawberry dataset information visualization.

The collection time is not fixed, and images are randomly captured under different lighting conditions. A total of 4970 strawberry images at 1640 pixels × 720 pixels were obtained. These images include various shapes and maturity levels of strawberries, taking into account factors like occlusion and overlap. Fruits were photographed from multiple angles to ensure a diverse sample set. In order to assess the effectiveness of the YOLOv9 algorithm, the image dataset was randomly partitioned into three groups, the model training set, verification set, and test set, with ratios of 7:2:1, respectively. These datasets will be utilized for model training, parameter optimization, and the comparison of prediction results to evaluate the model’s performance. The visualization in Figure 15 presents the strawberry dataset. In Figure 15A, the types and corresponding label information are displayed, with the green ripening period is labeled as A, white ripening period as B, color-changed period as C, and red ripening period as D. Figure 15B illustrates the dimensions of the label box, while Figure 15C shows the distribution of center-point locations. Figure 15D provides information on the distribution of strawberry sizes, and Figure 15E assigns details to the labels.

The model training and testing environments for this study are Intel (R) Xeon (R) 8350C CPU, RTX3090 GPU, 24GB RAM, Python 3.8 deployment environment, Python 1.11.0 deep learning framework, CUDA 1.3 acceleration environment, input image size of 640 × 640, batch size of 64, initial learning rate of 0.01, weight attenuation coefficient of 0.0005, and an SGD optimizer, with optimizer Momentum set to 0.937.

### 3.2. Model Evaluation Indicators

When deploying a lightweight strawberry ripeness detection model at the edge, it is crucial to consider both the accuracy of the detection and the complexity of the model. This article evaluates model performance using metrics such as precision, recall, mAP@50%, mAP@50:95%, and frames per second. Precision is defined as the ratio of correct predictions to predicted positive samples, while recall is the ratio of correct samples predicted by the model to the total samples. mAP is commonly measured using two indicators: mAP@50% and mAP@50:95%. The former calculates the average accuracy mean at an IoU threshold of 0.5, while the latter measures the average mAP across ten groups with IoU values ranging from 0.5 to 0.95 at increments of 0.05.

### 3.3. Model Performance Experiments

**Figure 16:**
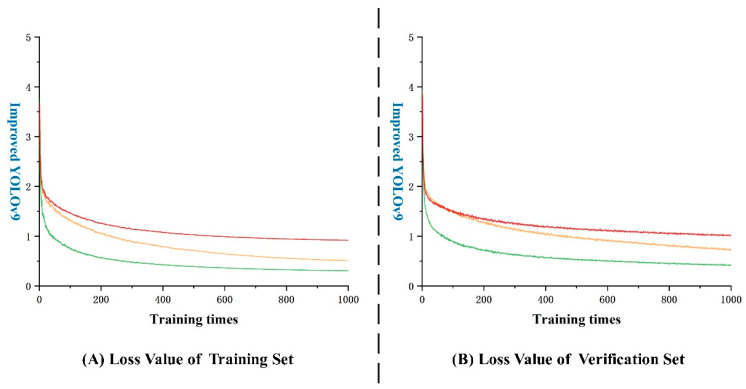
Loss function curve (note: yellow: boundary frame loss; green: classification loss; red: feature point loss).

The performance of a model can be evaluated by the loss function, with a smaller value indicating a better alignment with real results. In the case of CR-YOLOv9 shown in Figure 16A, a rapid decrease in the loss function is observed initially during training. However, after around 200 rounds, the rate of decrease slows down and the curve starts to noticeably oscillate. By 600 rounds, the model’s loss function stabilizes, with bounding box, classification, and feature point losses in the training set settling below 1.2. In Figure 16B, a similar pattern is seen with CR-YOLOv9, where after 300 rounds, the decrease rate slows down, leading to more pronounced oscillations. After 600 rounds, the model’s loss function stabilizes, with losses in the training set remaining below 1.5.

### 3.4. Ablation Experiment

**Figure 17:**
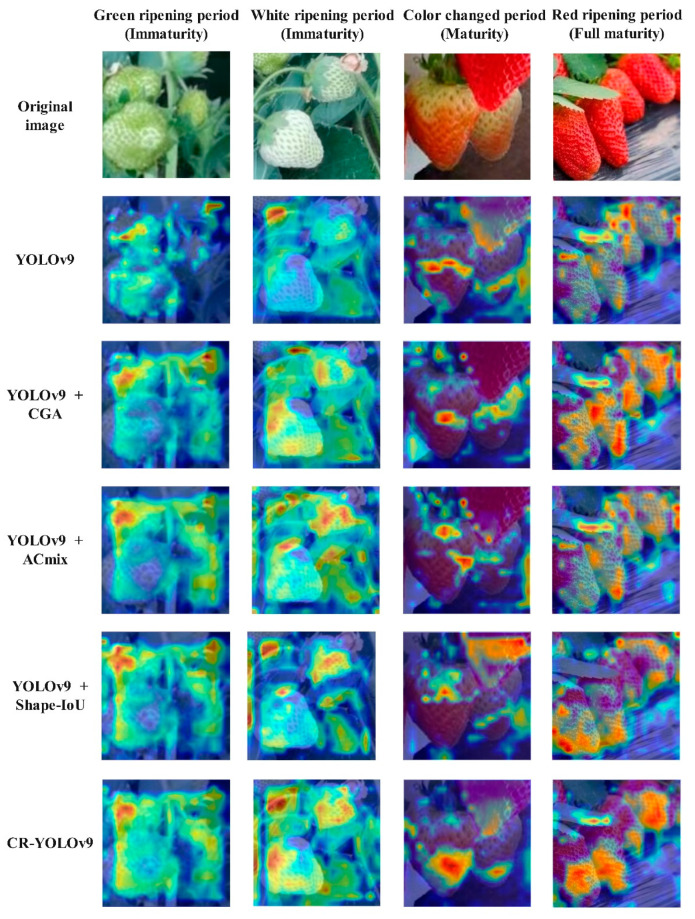
Grad CAM thermal map results for different improved modules.

In order to assess the impact of three enhancements on the model’s performance, YOLOv9 was chosen as the baseline model. Ablation tests were carried out on the test set of a custom dataset. The Grad-CAM heat map outcomes for the various enhancement modules are depicted in Figure 17, while the test results are summarized in Table 2.

Ablation experiments demonstrate that the proposed enhanced methods lead to improved detection performances across various metrics. The subsequent experiments reveal that integrating the CGA module effectively reduces the computational redundancy and enhances network depth, thereby boosting the model’s capacity. Furthermore, the ACmix approach combines the combined convolution network and self-attention module to streamline computational overheads and enhance lightweight aggregation operations. Additionally, the Shape-IoU method significantly improves the model’s generalization ability when confronted with varying strawberry shapes. In comparison to the original model, the CR-YOLOv9 model introduced in this study shows enhancements in accuracy by 4.2%, recall rate by 5.07%, mAP@50% by 3.34%, mAP@50:95% by 16.64%, and F1 value by 4.65%. Notably, the detection speed remains at 84, meeting the real-time detection requirements and facilitating improved strawberry maturity detection in greenhouse settings.

### 3.5. Comparison of Mainstream Algorithms

**Figure 18:**
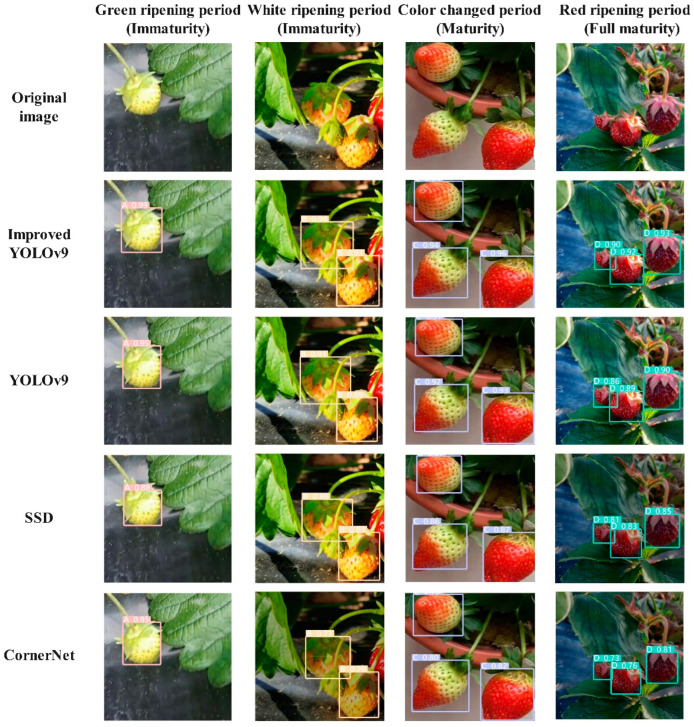
Comparison of detection results of different models.

The CR-YOLOv9 model, alongside the original YOLOv9 model [47] and two mainstream target detection models, SSD [48] and CornerNet [49], underwent training and verification in a consistent experimental environment. The analysis of the detection results across different models is illustrated in Figure 18, with precision, recall, mAP50%, mAP50:95%, F1 Score%, and FPS serving as key performance indicators. The experimental findings, detailed in Table 3, demonstrate that the CR-YOLOv9 model outperforms the other three mainstream models in terms of the detection accuracy and speed.

Table 3 illustrates that SSD and CornerNet exhibit poor detection effects, whereas YOLOv9 and CR-YOLOv9 demonstrate better performances. Specifically, CR-YOLOv9 achieves an average precision that is 4.2%, 9.75%, and 16.55% higher than YOLOv9, SSD, and CornerNet, respectively. Furthermore, its recall rate surpasses CornerNet by 21.07% and YOLOv9 by 5.07%. In terms of FPS, CR-YOLOv9 shows a reduction of 10 and 8 frames compared to SSD and CornerNet, respectively. These results suggest that CR-YOLOv9 effectively enhances real-time detection accuracy without imposing a significant computational burden, making it suitable for practical area scene detection.

## 4. Conclusions

This study focuses on addressing the challenges associated with detecting strawberry ripeness in a greenhouse environment. Based on the latest model, YOLOv9, of the YOLO family, a CR-YOLOv9 multi-stage algorithm is introduced for strawberry fruit maturity detection. This algorithm aims to improve strawberry picking efficiency and reduce manual labor compared to traditional production methods, while also addressing issues such as high costs and suboptimal picking times.

1. Conducting a comprehensive collection of multi-stage strawberry maturity image datasets in a controlled greenhouse setting is essential to guarantee the accuracy and credibility of the experimental data.

2. The composite thinning network CRNet is introduced, which utilizes multi-branch blocks for target fusion to restore high-frequency details in the image and enhance overall image quality. The design concept of CGA is incorporated to align input feature grouping with the number of attention heads, providing distinct segmentations of complete features for each attention head. This approach explicitly decomposes attention calculations across the heads. Additionally, a hybrid operator, ACmix, is proposed to reduce computational complexity and enhance image quality by efficiently sharing self-attention and convolution modules. The introduction of the Inner-IoU concept, combined with Shape-IoU (shape intersection over union), replaces the original loss function. This modification directs the model to focus more on recognizing overlapping detection frames, thereby improving the accuracy of detecting small targets in various scenarios.

3. A plethora of ablation experiments and comparative results demonstrates that CR-YOLOv9 significantly enhances the accuracy of multi-stage strawberry ripeness detection while maintaining a relatively fast detection speed. The precision rate, recall rate, and average precision of CR-YOLOv9 achieved 93.32%, 90.27%, and 94.61%, respectively, marking an improvement of 4.2%, 5.07%, and 3.34%, respectively, compared to the original YOLOv9 model.

The next step is to enhance the accuracy of multi-stage strawberry maturity using multi-modal methods, deploy lightweight edge devices, establish a comprehensive management and traceability system ranging from planting source to market sales, improve the organization and management of the strawberry industry, and achieve green and sustainable developments in agriculture and the food industry. This will promote a virtuous cycle of economy and society.

## Figures and Tables

**Table 1:** Classification description of strawberry maturity.

Stage	Maturity	Related Description	Picking Situation
1	Green ripening period	During the early stages of strawberry growth, the fruit appears bright green in color with a smooth skin and firm flesh. At this point, the fruit lacks the characteristic red or white spots and is not yet sweet in taste.	Not suitable for picking
2	White ripening period	During the middle stage of strawberry growth, the majority of the fruit surface appears white as the green color begins to fade, although some green areas may still be present. At this point, the pulp is soft, but has not yet reached optimal sweetness.	Not suitable for picking
3	Color-changed period	During the later stages of strawberry growth, the fruit typically displays a noticeable red color. This color change typically starts on the side of the fruit that receives the most light, before gradually spreading to the other areas including the sides and backlit surface.	Pickable, stored, or transported over long distances
4	Red ripening period	During the final stage of strawberry growth, the fruit undergoes a transformation, turning a vibrant red color with a uniform appearance, free of any green or white spots. As the fruit reaches maturity, it also tends to increase in size and develop a sweeter taste.	Pickable, transported at close distances, or sold on the same day

**Table 2:** Results of ablation experiments.

Model	Precision *P*/%	Recall *R*/%	Mean Average Precision mAP@50/%	Mean Average Precision mAP@50:95/%	*F*1 Score/%	Frames per Second FPS/(Frame·s^−1^)
YOLOv9	93.32	90.27	94.61	68.82	91.77	78
YOLOv9 + CGA	94.57	91.39	95.36	72.12	92.96	86
YOLOv9 + ACmix	94.78	90.67	95.19	71.01	92.68	84
YOLOv9 + Shape-IoU	95.12	90.92	94.97	71.94	92.97	79
YOLOv9 + CGA + ACmix	95.71	92.52	96.01	76.19	94.09	80
YOLOv9 + CGA + Shape-IoU	95.22	92.58	95.94	74.69	93.88	81
YOLOv9 + ACmix + Shape-IoU	95.34	92.15	95.72	73.49	93.71	83
YOLOv9 + CGA+ ACmix+Shape-IoU	97.52	95.34	97.95	85.46	96.42	84

**Table 3:** Comparison of experimental results.

Model	Precision *P*/%	Recall *R*/%	Mean Average Precision mAP@50/%	Mean Average Precision mAP@50:95/%	*F*1 Score/%	Frames per Second FPS/(Frame·s^−1^)
CR-YOLOv9	97.52	95.34	97.95	85.46	96.42	84
YOLOv9	93.32	90.27	94.61	68.82	91.77	78
SSD	87.77	82.73	88.13	55.16	85.17	94
CornerNet	80.97	74.27	81.56	47.72	77.48	92

## Data Availability

The original contributions presented in the study are included in the article. Further inquiries can be directed to the corresponding authors.
